# Influence of sagittal root positions on the stress distribution around custom-made root-analogue implants: a three-dimensional finite element analysis

**DOI:** 10.1186/s12903-021-01809-4

**Published:** 2021-09-14

**Authors:** Chunping Lin, Hongcheng Hu, Junxin Zhu, Yuwei Wu, Qiguo Rong, Zhihui Tang

**Affiliations:** 1 Department of Periodontology, Peking University School and Hospital of Stomatology, National Engineering Laboratory for Digital and Material Technology of Stomatology, Beijing Key Laboratory of Digital Stomatology, Beijing, 100081 China; 2 Second Dental Center, Peking University School and Hospital of Stomatology, National Engineering Laboratory for Digital and Material Technology of Stomatology, Beijing Key Laboratory of Digital Stomatology, Beijing, 100101 China; 3 Department of Mechanics and Engineering Science, College of Engineering, Peking University, Beijing, 100871 China

**Keywords:** Root-analogue implant, Sagittal root position, Stress concentration

## Abstract

**Background:**

Stress concentration may cause bone resorption even lead to the failure of implantation. This study was designed to investigate whether a certain sagittal root position could cause stress concentration around maxillary anterior custom-made root-analogue implants via three-dimensional finite element analysis.

**Methods:**

The von Mises stresses in the bone around implants in different groups were compared by finite element analysis. Six models were constructed and divided into two groups through Geomagic Studio 2012 software. The smooth group included models of unthreaded custom-made implants in Class I, II or III sagittal root positions. The threaded group included models of reverse buttress-threaded implants in the three positions. The von Mises stress distributions and the range of the stresses under vertical and oblique loads of 100 N were analyzed through ANSYS 16.0 software.

**Results:**

Stress concentrations around the labial lamella area were more prominent in the Class I position than in the Class II and Class III positions under oblique loading. Under vertical loading, the most obvious stress concentration areas were the labial lamella and palatal apical areas in the Class I and Class III positions, respectively. Stress was relatively distributed in the labial and palatal lamellae in the Class II position. The maximum von Mises stresses in the bone around the custom-made root-analogue implants in this study were lower than around traditional implants reported in the literature. The maximum von Mises stresses in this study were all less than 25 MPa in cortical bone and less than 6 MPa in cancellous bone. Additionally, compared to the smooth group, the threaded group showed lower von Mises stress concentration in the bone around the implants.

**Conclusions:**

The sagittal root position affected the von Mises stress distribution around custom-made root-analogue implants. There was no certain sagittal root position that could cause excessive stress concentration around the custom-made root-analogue implants. Among the three sagittal root positions, the Class II position would be the most appropriate site for custom-made root-analogue implants.

## Background

Today, patients typically seek aesthetic and convenient restorations. Immediate implantation with custom-made root-analogue implants (RAIs) in the aesthetic zone has gained much attentions in recent decades for its advantages over traditional implants [1].

RAIs are defined as implants that have the same contour as the extracted root [2, 3]. Compared to traditional implants, RAIs have some advantages, such as a better fit in the extraction socket, eliminating the need for conventionally used bone drills and other traumatic procedures that are required to prepare for implantation, as well as uncomplicated immediate implant placement, increased patient comfort, and better aesthetic effectiveness through immediate temporary restoration [1, 4–8].

Although many researchers had reported many successful clinical cases using RAIs in the past years [6, 9], there have been some failures [8, 10]. Research has shown that stress distribution in the bone around implants under loading is related to the success of implantation [11]. High stress can cause bone resorption and implant loosening, finally leading to the failure of implantation [12]. Therefore, it is necessary to predict the stress around RAIs to provide a prognosis for surgery preoperatively.

Upper incisors have different sagittal root positions [16–18]. In Kan’s research, the sagittal root positions of the upper incisors were classified as Class I, II, III, or IV [19]. In Class I, the root is positioned against the labial cortical plate. In Class II, the root is centered in the middle of the alveolar housing without engaging either the labial or the palatal cortical plates at the apical third of the root. In Class III, the root is positioned against the palatal cortical plate, and in Class IV at least two-thirds of the root engages both the labial and palatal cortical plates. Stress distribution in the peri-implant bone can be different when RAIs are placed in different sagittal root positions.

The influence of sagittal root position is quite different between traditional implants and RAIs. Traditional implant may be implanted in a position that is completely different from the initial sagittal root position of the extracted tooth [13]. For example, when the extracted tooth is in the Class I position and a traditional implant is used to complete the immediate implantation, the implant may be positioned toward the palatal side to take advantage of more supporting bone [14, 15]. However, after implantation, the sagittal root positions of RAIs are congruent with the extracted roots because RAIs are implanted along the extraction socket [20].

To promote the success of implantation with RAIs in the anterior region, it is necessary to clarify the influence of different sagittal root positions of upper incisor RAIs on peri-implant bone stress distributions. If there is a certain sagittal root position that could cause high stress concentrations around RAIs, measures should be undertaken to avoid bone resorption. However, until now, there have been few studies that could provide a predicable prognosis for RAI surgery.

Thus, this study aimed to investigate the influence of different sagittal root positions of maxillary central incisor RAIs on peri-implant bone stress distribution by means of finite element analysis (FEA).

## Methods

### Construction of the anterior maxilla model

A cone-beam computer tomography (CBCT) image from a male patient, in which the dentition was sound and the labial and palatal bone lamellae were intact, was selected from a series of CBCT images obtained from patients who visited the Second Clinical Division, Peking University School and Hospital of Stomatology. It should be noted that this research was approved by the Biomedical Ethics Committees of Peking University School and the Hospital of Stomatology (approval number PKUSSIRB-201734034) in accordance with the 1964 Helsinki declaration. At the same time, in this study, written informed consent was obtained from all subjects or, if subjects are under 18, from a parent and/or legal guardian. The CBCT image was stored in digital imaging and communications in medicine (DICOM) format and imported into the medical image processing software (Mimics,version 18.0; Materialise, Leuven, Belgium). The thickness of the CBCT data was 0.15 mm. According to the threshold differences between the tooth and the alveolar bone (the threshold of the grey values of the tooth in the Mimics software was 1445-3978, while that of the alveolar bone was 959-1445), the tooth and the alveolar bone were segmented preliminarily. Then the two different tissues were segmented finely by hand through naked eye. The upper right incisor model and a corresponding bone model were constructed and saved in standard tessellation language (STL) format.

In accordance with the method described by Chen et al.[15], the thicknesses of both the labial and palatal cortical bones were measured at 5 different sites, and the average thicknesses (0.8 and 1.2 mm, respectively) were calculated. The STL format data files of the upper right incisor and the corresponding bone model were then entered into reverse engineering software (Geomagic Studio 2012; Raindrop Geomagic, Research Triangle Park, NC, USA) to obtain a solid model.

**Fig. 1:**
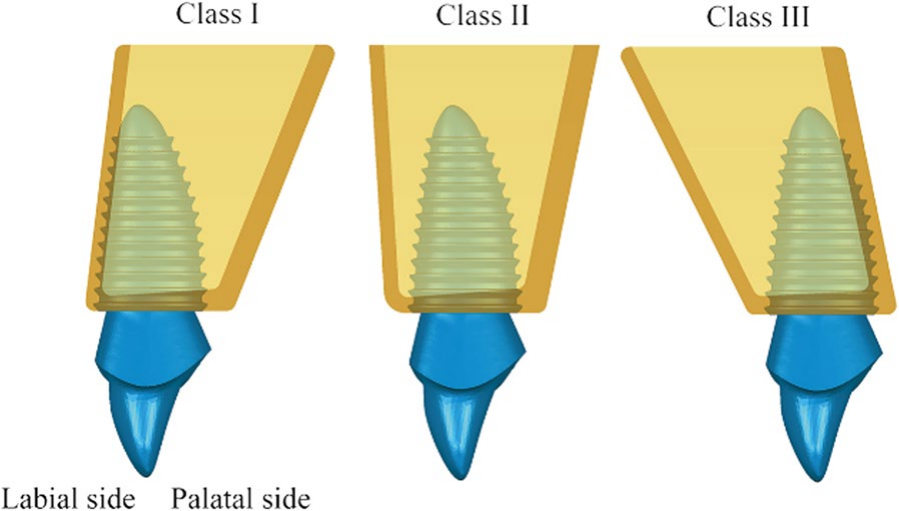
Models of RAIs in different sagittal root positions of the maxillary central incisor. (Class I: the root is positioned against the labial cortical plate. Class II: the root is centered in the middle of the alveolar housing without engaging either the labial or the palatal cortical plates at the apical third of the root. Class III: the root is positioned against the palatal cortical plate.)

The upper alveolar bone model was modified using Siemens NX software, version 12 (Siemens PLM Software, Berlin, Germany), to obtain simplified anterior maxillary bone block models when the upper right incisor was in the Class I, II, and III sagittal root position. An alveolar ridge width of 7 mm was maintained, with an alveolar ridge crest angle of 15°, a labial bone thickness of 0.8 mm, and a palatal bone thickness of 1.2 mm for the three bone models (Fig. 1).

### Construction of the implant models

**Fig. 2:**
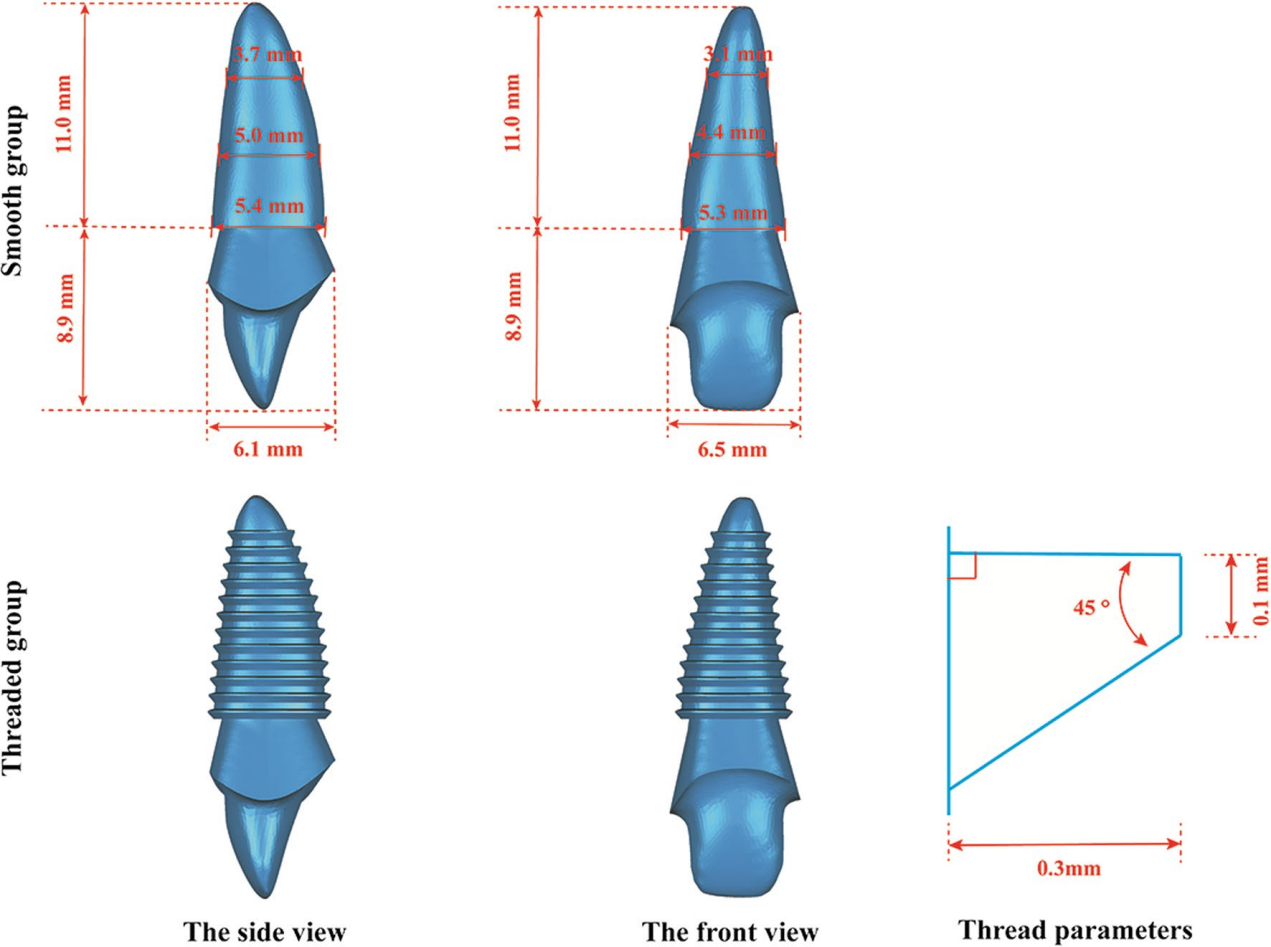
Different RAI models and detailed parameters. The diameters in the cervical, middle, and apical thirds in the threaded group are 0.3 mm larger than the smooth group

Based on the previously constructed tooth model, a one-stage root-analogue implant model with a custom-made abutment and the corresponding restoration crown were designed using Geomagic Studio 2012 software. The margin of the custom-made abutment was designed along the cementum-enamel junction (CEJ). The shape of the shoulder was designed to have a similar right angle, while its inner angle was obtuse. The width of the shoulder was 1 mm. The corresponding all-ceramic crown was then designed based on the shape of the original tooth. RAI models without threads were designated as the smooth group. Next, a reverse buttress thread design was added to the surface of the implant; these models were designated the threaded group. Their shapes and detailed parameters are shown in Fig. 2.

Different alveolar bone models were then combined with RAI models with different thread designs. Six root-analogue implants and alveolar bone groups were obtained that included all of the conditions of implants with different surface designs (smooth and threaded) in different sagittal root positions. The positions of RAIs for different classes are shown in Table 1. The labial-palatal distances from the outermost point in the cervical region of the implant to the surface of the cortical bone on both labial side and palatal side were measured. In addition, the distances from the implant apical point to the surface of the cortical bone on both labial side and palatal side were also measured.

**Table 1:** The positioning distances of RAIs in different sagittal root positions

Sagittal root position	Smooth/threaded group	Labial/palatal side	Labial-palatal distances (mm)	
			In the cervical region	In the apical region
Class I	Smooth group	Labial side	0.7	1.3
		Palatal side	1.8	10.4
	Threaded group	Labial side	0.4	1.3
		Palatal side	1.5	10.4
Class II	Smooth group	Labial side	1.6	4.5
		Palatal side	1.6	7.8
	Threaded group	Labial side	1.3	4.5
		Palatal side	1.3	7.8
Class III	Smooth group	Labial side	1.3	9.9
		Palatal side	1.2	2.0
	Threaded group	Labial side	1.0	9.9
		Palatal side	0.9	2.0

### Elements and nodes

Previously developed models were imported into Hypermesh software, version 10.0 (Altair Engineering, Troy, MI, USA), to mesh with 10-node tetrahedron elements (285 solid elements). The smooth implant model comprised 746,339 elements and 133,091 nodes in Class I position, 579,088 elements and 104,623 nodes in Class II position, 734,900 elements and 131,476 nodes in Class III position separately. The threaded implant model comprised 932,232 elements and 165,016 nodes in Class I position, 1,050,500 elements and 185,101 nodes in Class II position, 984,867 elements and 174,297 nodes in Class III position separately. All of the data were stored in fast database dump (cdb) format and then imported into ANSYS software, version 16.0 (Ansys, Canonsburg, PA, USA).

Similar to Roy’s research [16], mesh convergence tests were performed to ensure the accuracy of the numerical results.

### Interface conditions

The following assumptions were made for these finite element models: the implant-bone and implant-crown interfaces were assumed to be in bonded contact.

### Material properties

All of the materials used in the models were considered isotropic, homogeneous, and linearly elastic. All of the material parameters were based on similar previous studies [15, 17–19] (Table 2).

**Table 2:** Summary of the material properties used for the finite element analysis

Materials	Young’s modulus E (GPa)	Poisson’s ratio ν
Titanium	110.00	0.30
Cortical bone	13.70	0.30
Cancellous bone	1.37	0.30
Porcelain	70.00	0.19

### Constraints and loading conditions

All of the nodes from the mesial, distal, and bottom surfaces of the alveolar bone models (including cortical and cancellous bone) were selected, and the degrees of freedom in the x, y, and z directions were set to 0.

**Fig. 3:**
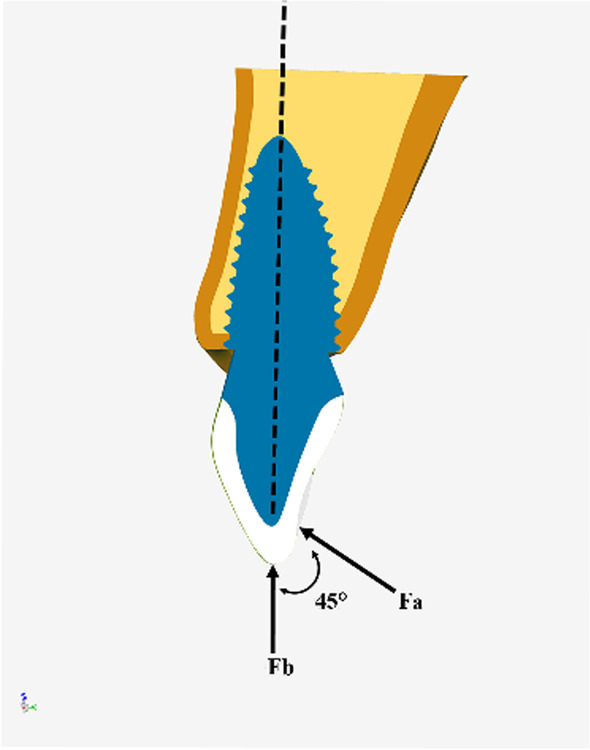
Schematic representation of loadings (Black vectors indicate the direction of the applied vertical and oblique forces: Fa, Oblique loads; Fb, Vertical loads)

To simulate a upper incisor during chewing movement [20], two loads were applied to the RAI finite element models, as shown in Fig. 3.

1. Oblique load (simulating the central occlusion): the load magnitude was 100 N, the load direction was 45° to the long axis of the implant, and the load position was on the lingual fossa of the crown.
2. Vertical load (simulating the edge-to-edge occlusion): the load magnitude was 100 N, the load direction was parallel to the long axis of the implant, and the load position was on the central area of the incisor edge of the crown.

### Analysis

The six finite element models were imported into ANSYS software, version 16.0, to compare the von Mises stress distributions of the peri-implant bone in different surface designs (smooth and threaded) and different sagittal root positions. In addition, the range of von Mises stresses in the bone tissue, namely in the region from alveolar crest to the area 5 mm below it.

## Results

### Distributions of von Mises stress

**Fig. 4:**
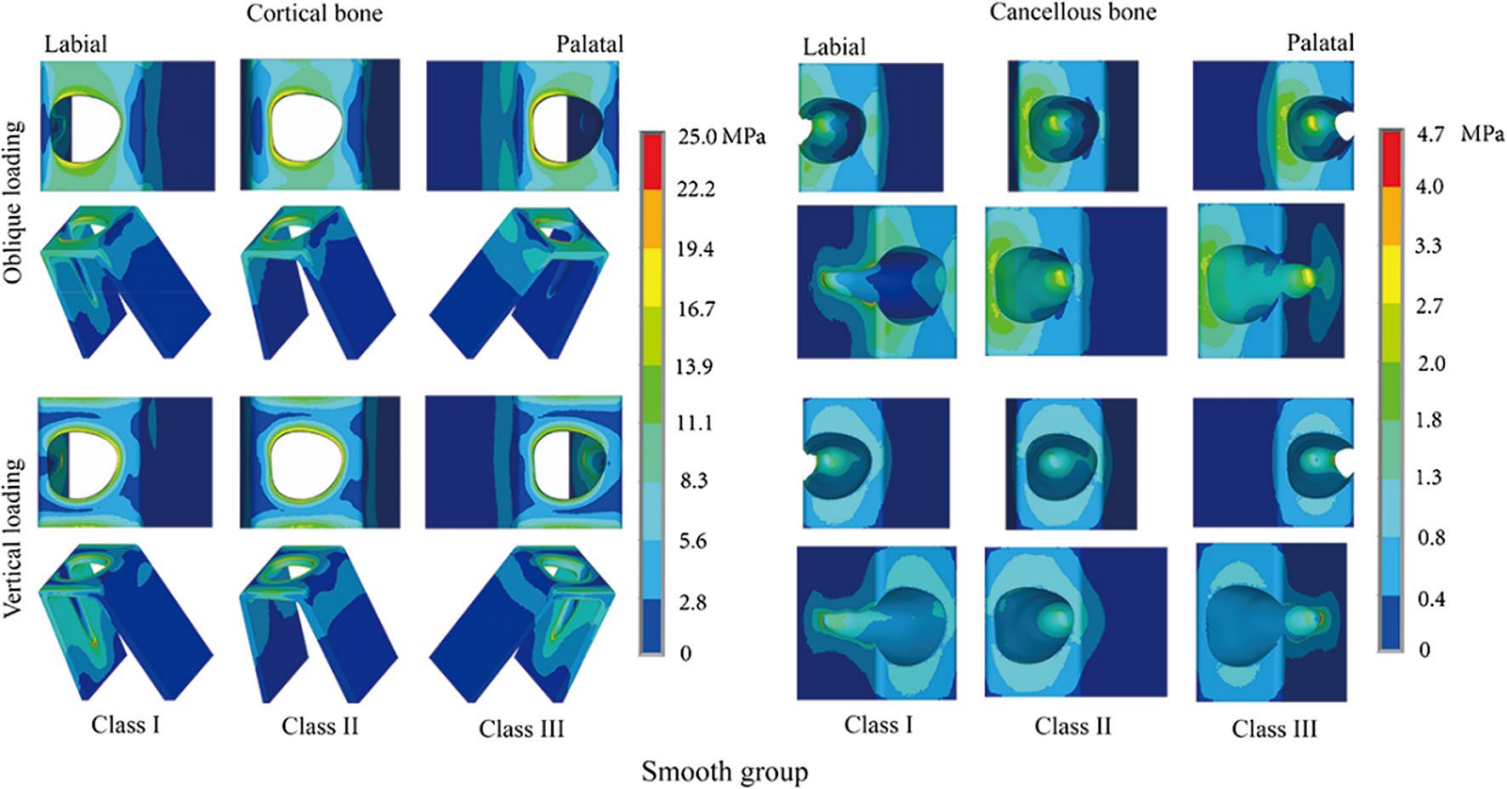
Distribution patterns of von Mises stress around RAIs with no thread design in different sagittal root positions

The distribution patterns of von Mises stress around RAIs in different sagittal root positions in the smooth group are shown in Fig. 4. Under oblique loading, the von Mises stress in the cortical bone was particularly notable in the labial crest area around the implant neck, the labial lamella, and the apical areas. Stress concentration was most apparent in labial areas in the Class I position. The implant neck areas in the Class II and Class III positions exhibited greater stress concentrations than those in the Class I position. Similarly, von Mises stress in the cancellous bone also indicated high stress concentrations in the labial crest area around the implant neck, the labial lamella, and the implant apical area. Stress in the implant labial neck area and apical area was more apparent in the Class II and Class III positions than in the Class I position.

Under vertical loading, von Mises stress mainly appeared in the implant neck area and apical area. Stress concentrations in the cortical bone around the implant neck area were least notable in the Class III position. The most apparent stress concentration area was in the labial lamella in the Class I position, while it was in the palatal apical area in the Class III position. Stress was relatively distributed in the labial and palatal lamellae in the Class II position. The von Mises stress distribution in cancellous bone was similar in the three sagittal root positions. Stress was mainly concentrated in the alveolar bone crest area around the implant neck and apical areas. The highest stress level in the apical area was observed in the Class I position, while the lowest stress concentration in the cancellous bone around the implant neck area was observed in the Class III position. The stress concentration around the implant neck area was particularly notable in the Class II position.

**Fig. 5:**
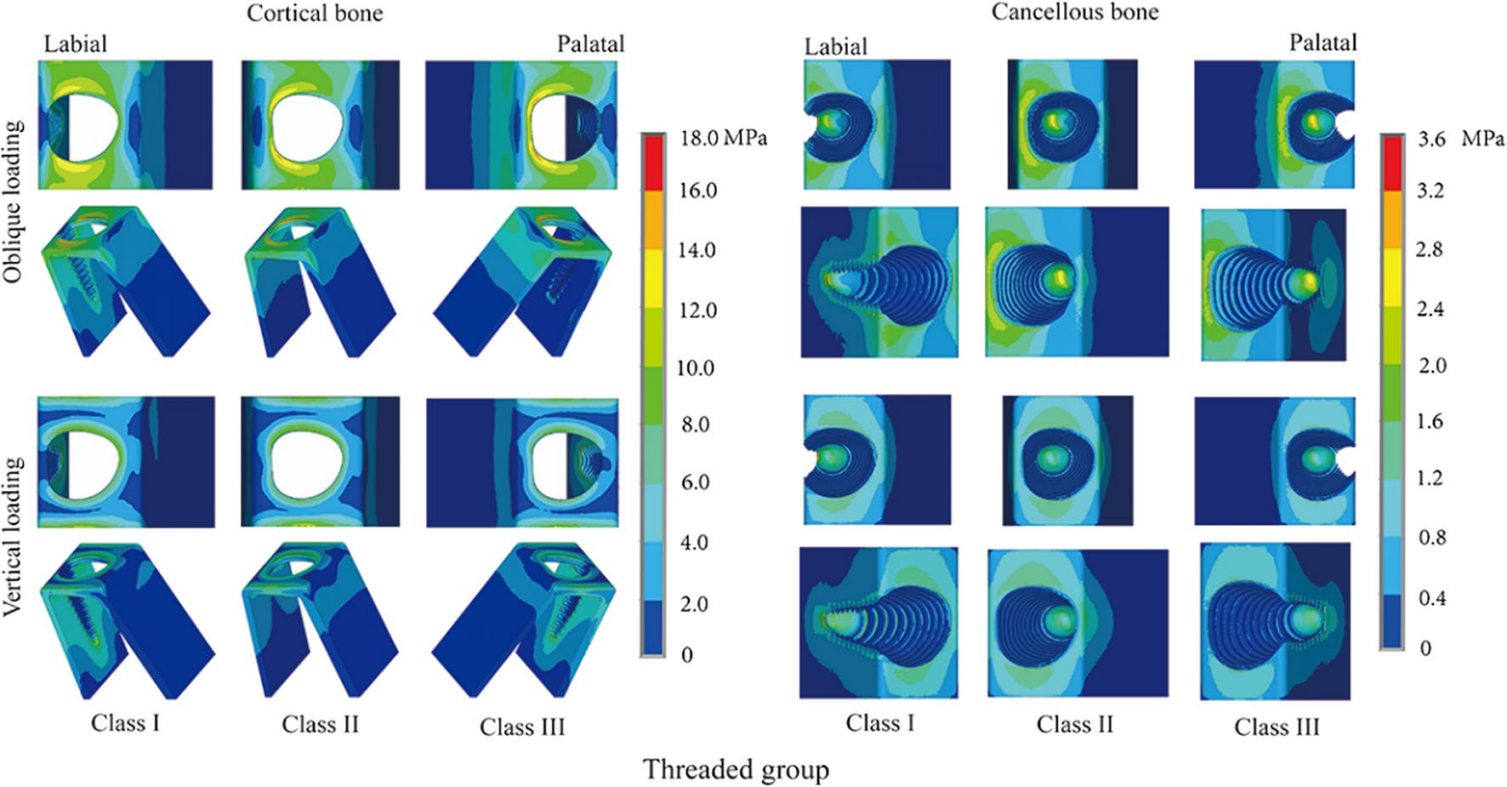
Distribution patterns of von Mises stress around RAIs with reverse buttress thread design in different sagittal root positions

The von Mises stress concentration conditions around RAIs in the threaded group in different sagittal root positions are compared in Fig. 5. Differences in stress concentration conditions among the three sagittal positions were more prominent in the threaded group, especially under oblique loading. The implant labial neck area in the Class III position exhibited the greatest stress concentration in the cortical bone. When in the cancellous bone, the highest stress level in the neck area was observed in the Class II position, while in the apical area it was observed in the Class III position. Differences in the stress distribution conditions among the different sagittal positions under vertical loading were not as apparent as under oblique loading. The von Mises stress distributions in the neck regions were similar in cortical bone; however, there was more apparent stress concentration in the labial and palatal lamella for the Class I and Class III positions. The stress concentration around the implant neck area was more notable in the Class II position than in the other two sagittal positions in the cancellous bone.

**Fig. 6:**
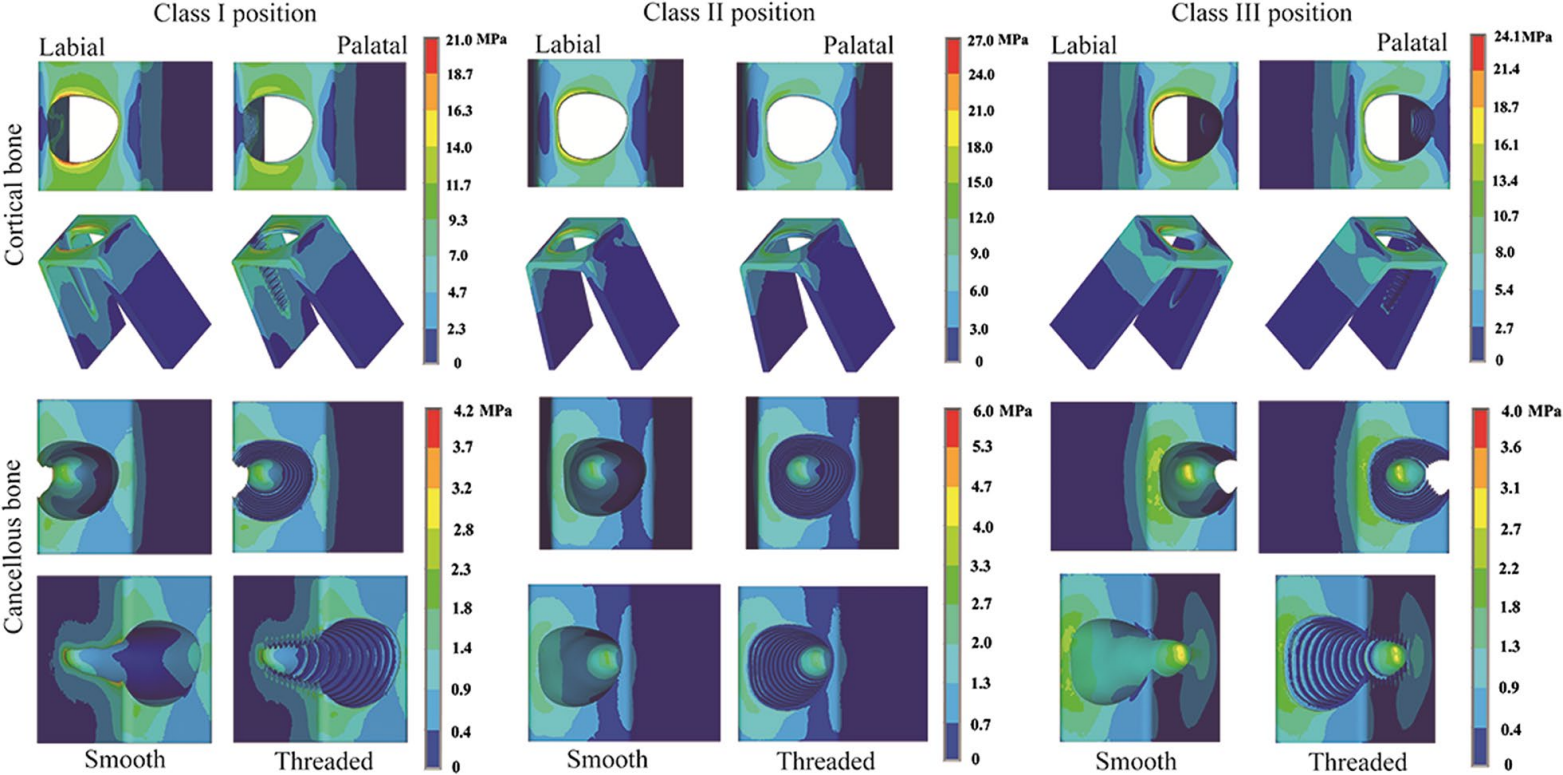
Distribution patterns of von Mises stress around RAIs under oblique loading in different sagittal root positions

**Fig. 7:**
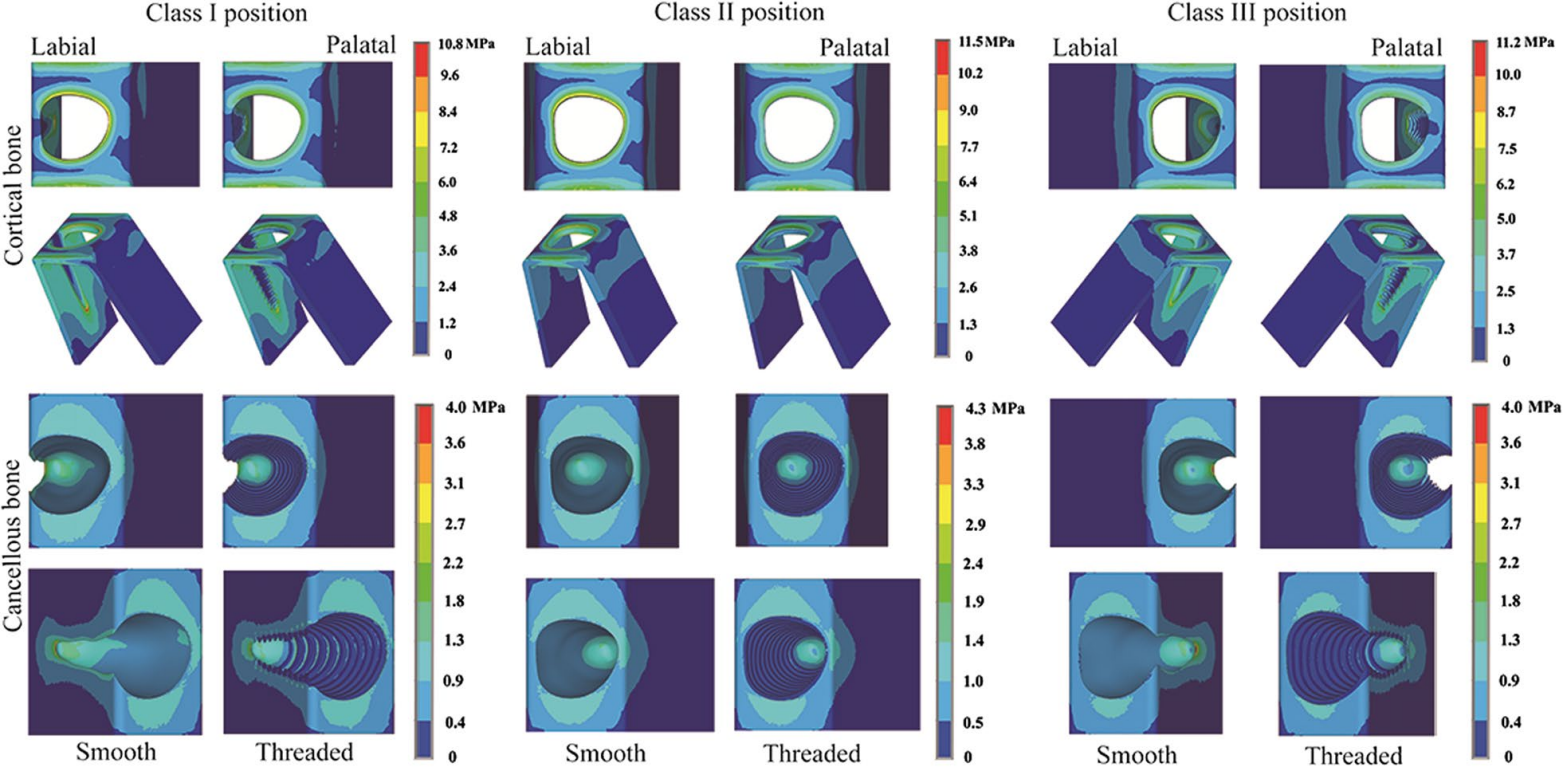
Distribution patterns of von Mises stress around RAIs under vertical loading in different sagittal root positions

Comparing the stress distributions between the smooth group and the threaded group (Figs. 6 and 7) revealed that the regional stress concentrations were similar when the implants were in the same sagittal root position. In addition, regardless of the loading pattern, whether in cortical bone or cancellous bone, the von Mises stresses in bone around implants with a reverse buttress thread design were all lower than those in bone around implants without threads.

### Ranges of von Mises stress

The range of von Mises stresses in the cortical and cancellous bone under different loadings in smooth group and threaded group were shown in Tables 3 and 4 separately.

**Table 3:** The range of von Mises stresses in the cortical and cancellous bone in the smooth group

Sagittal root position	Bone type	The range of von Mises stresses (MPa)			
		Oblique loading		Vertical loading	
		Maximum	Minimum	Maximum	Minimum
Class I	Cortical bone	20.83	0.18	9.16	0.31
	Cancellous bone	3.66	0.06	1.58	0.11
Class II	Cortical bone	24.91	0.11	10.02	0.22
	Cancellous bone	3.32	0.02	1.65	0.14
Class III	Cortical bone	24.12	0.02	7.80	0.18
	Cancellous bone	2.82	0.01	1.20	0.11

**Table 4:** The range of von Mises stresses in the cortical and cancellous bone in the threaded group

Sagittal root position	Bone type	The range of von Mises stresses (MPa)			
		Oblique loading		Vertical loading	
		Maximum	Minimum	Maximum	Minimum
Class I	Cortical bone	17.98	0.55	8.88	0.15
	Cancellous bone	3.44	0.02	2.15	0.02
Class II	Cortical bone	15.83	0.12	10.94	0.20
	Cancellous bone	3.58	0.02	2.45	0.03
Class III	Cortical bone	16.13	0.05	9.97	0.16
	Cancellous bone	2.86	0.01	1.93	0.03

## Discussion

To promote the success of implantation with RAIs in the anterior region, any disadvantageous factors, for example, excessive stress concentration, should be avoided [21, 22]. Upper incisors have different sagittal root positions in the alveolar bone. However, whether a certain sagittal root position can lead to stress concentration in peri-implant bone when RAIs are under loading remains unknown. This study was designed to analyse the stress around RAIs in different sagittal root positions according to Kan’s classification to provide a prognosis for surgery preoperatively.

As stated by Kan, the Class IV position was unsuitable for immediate implantation because there was a limited amount of bone with which appropriate implant stability could be obtained after tooth extraction [14]. Therefore, we evaluated only Class I, II, and III sagittal root positions in this study.

The labial lamella in the upper anterior region is thin [23]. Bone resorption in the labial and neck cortical bone areas around the implant could be more likely to occur when the implant is exposed to excessive stress [24]. The results showed that the sagittal root position affects the von Mises stress distribution in the peri-implant bone. Among the three sagittal root positions, stress concentration was most apparent in the labial cortical bone area for implants in the Class I position, both under oblique loading and vertical loading. The reason was that, RAIs in the Class I position contacted the labial cortical bone directly. Then, when the stress distribution around the implant neck area was analyzed, stress concentration was most evident in the Class III position. At the same time, stress concentration in the palatal lamella was also most evident in the Class III position. It was the result from that the cortical bone directly contacted RAIs in the palatal apical area, when implants were in the Class III position. When implants were under oblique loading, they tended to show a rotational movement. The center of rotation of implants in the Class I and Class II positions might be located in the middle of the root, while the center might be in the apical region in the Class III position. Hence, there was a greater rotation amplitude for implants in the Class III position. That was the reason why the implant labial neck area in the Class III position exhibited the greatest stress concentration in the cortical bone, when implants were under oblique loading.

Bone resorption is one of the key factors affecting the success of implantation [25]. The higher that the stress concentration occur in the bone around the implant is, the higher the risk of bone resorption is [26]. Thus, with respect to the stress concentrations in the labial lamella and neck areas around implants, among the three sagittal root positions, the Class II position is better suited for immediate implantation with RAIs. This finding is different from those with traditional implants. When applying traditional implants to complete the immediate implantation in the aesthetic area, the Class I position is thought to be the best position for implantation because it can provide sufficient bone on the palatal side for implantation. In this way, the initial stability of the implants is guaranteed [14, 27].

Although from this study, the Class II position was found to be the most appropriate site for applying RAIs in the maxillary anterior region, according to Kan’s research, there were only 6.5% of maxillary central incisors in the Class II sagittal root position [14]. Similarly, in Xu’s study, the percentage was only 4.4% [13]. Most of the maxillary central incisors were in the Class I position. To broaden indications for RAIs, measures should be obtained to reduce the stress concentration. It was found that, adding threads or a targeted press-fit geometry to the surface of RAIs could decrease the stress concentration in the peri-implant bone [15, 19, 35].

In this research, stress distribution conditions in the smooth group and the threaded group were also compared. In both the cortical bone and the cancellous bone, stress concentrations in peri-implant bone were lower for the threaded implants than for the smooth implants, regardless of the sagittal root position and the loading form. These findings were similar to those of studies exploring the influence of thread design on the stress distribution in peri-implant bone around traditional implants [28–30] and RAIs [15, 31]. The results of this study once again confirmed that a threaded design would result in a lower stress concentration in the alveolar bone around RAIs.

Besides, the range of von Mises stresses in the cortical and cancellous bone under different loadings in different groups were also analysed. For bone resorption may occur in the coronal regions more easily, we only analyse the range of von Mises stresses in the region from alveolar crest to the area 5 mm below it. According to the results of Tables 3 and 4, it could be found that the maximum von Mises stress in the cortical bone was lower in the threaded group than in the smooth group under oblique loading. Many previous studies have assumed maximum bone strength to be a biological limit to bone failure and activation of bone resorption [17, 32, 33]. In addition, it has been reported that overloading of cortical bone occurs when the maximum von Mises stress exceeds 25–28 MPa. Similarly, cancellous bone overloading will occur when the maximum von Mises stress exceeds 6 MPa [34]. According to the results of this study, it could be found that none of the maximum von Mises stresses in the cortical and cancellous bone exceeded the stress criterion. Hence, from the perspective of the range of von Mises stresses, there was no excessive stress concentration in peri-implant bone when RAIs were in these three sagittal root positions. In other words, all these three sagittal root positions included in this study were suitable for immediate implantation with RAIs.

This research could provide guidance for the clinical application of RAIs. It was found that all these three sagittal root positions included in this study were suitable for immediate implantation with RAIs while the Class II position would be best suited. If RAIs are placed in the Class I position, attention should be paid to the thickness of the labial lamella, and immediate oblique loading after implantation should be avoided. At the same time, measures such as adding a thread design to the implant could be undertaken to reduce the stress concentration on bone around RAIs. Furthermore, as promoted in other studies, the diameter of the implant next to the buccal cortical bone could be reduced to avoid fracture of the bony wall and pressure-induced bone loss [9, 35]. When implanted in Class II and Class III positions, the thickness of the alveolar bone in implant labial neck areas should be noted. In addition, when implanted in the Class III position, the thickness of the remaining alveolar bone in the palatal apical areas should be noted. Although in general, the soft and hard tissue on the palatal side was thicker than that on the labial side, excessive local stress should also be avoided to prevent bone resorption and even fenestration on the palatal side, which can lead to implantation failure [36, 37].

There are some limitations to this study. First, the study analyzed the stress distribution conditions after osseointegration, but the conditions could be more concerning in immediate implantation when osseointegration is not yet complete. In a future study, we will explore this problem. Second, the study only concerned the influence of sagittal root positions at the level of the stress distribution. Micromotion analysis is important for the implant’s initial stability, therefore, it will be considered in the future study. Third, the resorption of the alveolar ridge in post-extraction sites was not considered. We plan to further modify the alveolar bone model to achieve more accurate results. Fourth, according to Roy’s study [38], bone quality and quantity can play a major role during the analysis. In the future study, we will explore the role of bone quality also. Lastly, all results in this study should be validated clinically. The clinical researches are ongoing, and the relevant results will be reported in the future.

## Conclusions

The sagittal root position affected the von Mises stress distribution around RAIs. There was no certain sagittal root position that could cause high stress concentration around RAIs. Among the three sagittal root positions, the Class II position would be the most appropriate site for RAIs. Moreover, adding threads to the surface of implants is encouraged to reduce stress concentrations in the peri-implant bone.

## Data Availability

Data used to support the findings of this study are available from the corresponding authors upon request.

## Abbreviation

RAI: Root-analogue implant
